# Antigenic Targets of Patient and Maternal Autoantibodies in Autism Spectrum Disorder

**DOI:** 10.3389/fimmu.2019.01474

**Published:** 2019-07-19

**Authors:** Rut Mazón-Cabrera, Patrick Vandormael, Veerle Somers

**Affiliations:** Biomedical Research Institute, Faculty of Medicine and Life Science, Hasselt University, Diepenbeek, Belgium

**Keywords:** autism, immunoglobulins, brain antigens, neurodevelopement, placental transfer

## Abstract

Autism spectrum disorder (ASD) is a neurodevelopmental disorder whose behavioral symptoms become apparent in early childhood. The underlying pathophysiological mechanisms are only partially understood and the clinical manifestations are heterogeneous in nature, which poses a major challenge for diagnosis, prognosis and intervention. In the last years, an important role of a dysregulated immune system in ASD has emerged, but the mechanisms connecting this to a disruption of brain development are still largely unknown. Although ASD is not considered as a typical autoimmune disease, self-reactive antibodies or autoantibodies against a wide variety of targets have been found in a subset of ASD patients. In addition, autoantibodies reactive to fetal brain proteins have also been described in the prenatal stage of neurodevelopment, where they can be transferred from the mother to the fetus by transplacental transport. In this review, we give an extensive overview of the antibodies described in ASD according to their target antigens, their different origins, and timing of exposure during neurodevelopment.

## Introduction

Autism spectrum disorder (ASD) is a complex and highly heterogeneous neurodevelopmental disorder characterized by persistent deficits in social communication and social interaction (1). The patients also show restricted and repetitive patterns of behavior, interests and activities. Together with these symptoms, various comorbidities can occur, such as intellectual and language impairment, catatonia, epileptic seizures or attention-deficit/hyperactivity disorder (ADHD). These deficits can cause difficulties for the patient to understand, maintain or develop relationships, creating a barrier to their integration in social life.

ASD has become one of the most common neurodevelopmental disorders. The prevalence of ASD among 8-year-old children in the United States has more than doubled since 2000, increasing from 1 in 150 to 1 out of 59 children (2), and varies by sex, race/ethnicity and geographic area. It is four times higher among males than females and most common in non-Hispanic white children.

The diagnosis of ASD, usually made in early childhood, is based on observation of the atypical behaviors using questionnaires such as the Autistic Diagnostic Observation Schedule (ADOS) or Autism Diagnostic Interview-Revised (ADI-R). In each patient, the symptoms can have different manifestations caused by multiple etiologies, sub-types and developmental trajectories, often in combination with the different comorbidities, creating a complex condition (3).

ASD is considered a polygenic hereditary disorder, whose genetic component mostly comprises of common variant single-nucleotide polymorphisms (SNP) and copy number variations (CNV) which can occur both in coding and non-coding regions (4). Moreover, the presence of autistic behaviors in other monogenic disorders such as Angelman syndrome and Fragile X syndrome only shows part of the complexity of this developmental disorder (5). Besides a genetic predisposition, ASD is also strongly influenced by environmental factors, which mostly play their specific roles before, during and after pregnancy (6). Factors as diverse as parental age, prematurity, pollution or geographic area have been found to contribute to increased risk of ASD (2, 7–9).

An overarching understanding of the etiology of the disorder is still lacking, but many areas and processes in the brain have been implicated. Disturbances in activation and regulation of the posterior insula, limbic system or the cortico-striato-thalamo-cortical circuit, contribute to the picture of a complex disease, not restricted to a single anatomical location (10–12). At the cellular level, alterations in synaptogenesis, synaptic plasticity and pruning, add another dimension of complexity (13–15).

In the last decade, an important role of the immune system has been described in the development of ASD. The activation of microglia, the resident immune cells of the brain, and increased levels of pro-inflammatory cytokines in brain tissue, cerebrospinal fluid and blood give evidence for the presence of ongoing neuroinflammation in brains of some ASD patients (16–19). Moreover, *in utero* exposure to an inflammatory environment during pregnancy caused by maternal autoimmune disease or maternal immune activation by infections, is sufficient to impart lifelong neuropathology and altered behaviors in offspring (20, 21).

Although ASD and other neurodevelopmental disorders are not typically considered as autoimmune diseases, autoantibodies, or antibodies that bind to self-antigens, have been detected in the sera of patients with ASD, ADHD, bipolar affective disorder and schizophrenia (22). Autoantibodies against known targets have been linked to the development of behavioral symptoms in these disorders, for example anti-dopamine transporter autoantibodies have been related with severity and recovery in ADHD, antibodies against myelin basic protein (MBP) have higher titers in patients with negative symptoms in schizophrenia or N-methyl-D-aspartate receptor (anti-NMDAR) and glutamic acid decarboxylase (anti-GAD) antibodies have been associated with acute maniac episodes in bipolar disorder (23–25). The best-studied example are autoantibodies directed against the NMDAR in a condition known as anti-NMDAR encephalitis, characterized by the development of psychosis, cognitive problems and seizures. The pathogenic role of anti-NMDAR autoantibodies in this disorder has been demonstrated in cultured neurons and in animal models (26–28). Moreover, patients have even been shown to respond to immunotherapy (29).

**Figure 1:**
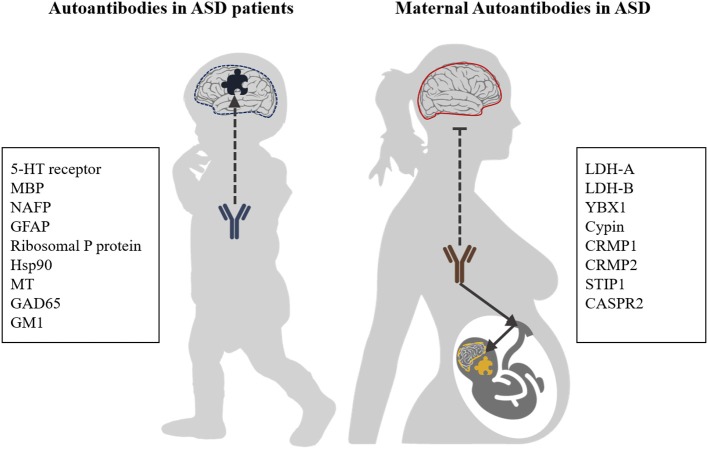
Autoantibody targets in ASD. Autoantibodies with reactivity to brain antigens are generated in a subpopulation of ASD patients. Furthermore, the process of transplacental transport of antibodies, which provides infants with passive immunity, can also transfer antibodies which are reactive against fetal brain antigens. It is generally assumed that the BBB of healthy mothers is intact and impermeable to (auto)antibodies, while for ASD patients increased BBB permeability has been described. 5-HT receptor, 5-hydroxytryptamine receptor; NAFP, Neuron-axon filament protein; GFAP, Glial fibrillary acidic protein; MBP, Myelin Basic Protein; GM1, Ganglioside M1; GAD65, Glutamic acid decarboxylase; Hsp90, Heat shock protein 90; MT, Metallothionein; LDH-A/B, Lactate dehydrogenase A and B; YBX1, Y-box-binding protein 1; Cypin, Cytosolic PSD-95 interactor; CRMP1, Collapsin response mediator proteins 1; CRMP2, Collapsin response mediator proteins 2; STIP1, Stress-induced phosphoprotein 1; and CASPR2, Contactin-associated protein-like 2.

Besides the formation of autoreactive antibodies in ASD patients themselves, such antibodies have also been described in the blood of mothers whose child will develop ASD (Figure 1) (30, 31). These maternal autoantibodies can be transferred from the mother to the child during pregnancy via transplacental transport and might play an etiological role in ASD as they can pass the immature fetal blood-brain barrier (BBB) which is actively changing and semi-permeable during neurodevelopment (32, 33).

In this review, we explore the current evidence of antibodies detected in ASD patients and in mothers of ASD patients, their possible role as biomarkers to support the disease diagnosis and their contribution in the understanding of disease mechanisms.

## Autoantibodies in ASD Patients

Autoantibodies in an ASD patient were first described by Todd et al. (31) in a study on hyperserotonemia. Antibodies in the blood and cerebrospinal fluid (CSF) of an ASD patient could block binding of radioactive serotonin to a human cortical brain homogenate. Although the presence of these autoantibodies was not supported by follow-up studies (34, 35), the idea that a subset of ASD patients may have antibodies against specific self-antigens in the brain started to gather much interest. Since then, autoantibodies in ASD patients have been mostly studied by looking at known, mostly brain-related autoantigens which have also been described in other autoimmune conditions (described below and in Table 1). Whether these specific autoantibodies can cross the BBB, bind to their antigens in the brain and contribute to disease worsening, has not been demonstrated experimentally so far. Nonetheless, an increased general permeability of the BBB has been described for several psychiatric disorders, including ASD [reviewed in Kealy et al. (52)] which could provide a mechanism that would allow these autoantibodies to reach the brain. Moreover, most of the autoantibody targets described in ASD patients are localized intracellularly. It has long been assumed that large biomolecules such as antibodies cannot traverse the plasma membrane. However, for some anti-DNA antibodies in systemic lupus erythematosus (SLE) patients, membrane translocation and nuclear targeting has been demonstrated (53). Still, for the ASD-related autoantibodies described below, such mechanisms have not been investigated.

**Table 1:** Overview of ASD patient autoantibodies.

**Autoantibodies in patients**									
**Antibody target**	**Isotype**	**MW (kDa)**	**Protein origin**	**Methods**	**Diagnosis**	**Sensitivity^a^**	**Specificity^b^**	**Cohort^c^**	**References**
Serotonin (5-HT) receptor	IgG	NR	Human	Equilibrium saturation	DSM-III	NR	NR	Case study	(31)
	IgG		Human	Ligand binding	DSM-III	NR	NR	3–19 years	(35)
Myelin basic protein (MBP)	IgG	14–21	Rabbit	Western blot	DSM-III	58% (19/33)	MR 85% (3/20) TD 78% (4/18) A 97% (1/38) DS 100% (0/12)	≤ 10 years, Adults 20–40 years	(36)
	IgG		Bovine	ELISA/Western blot	DSM-IV/ADOS/ADI-R	NR	NR	2–10 years	(37)
	IgG/ IgM		NR	ELISA	DSM-IV	NR	NR	2–9 years	(38)
	IgG		Human	ELISA	DSM-IV	80% (40/50)	TD 95% (1.5/30)	5–12 years	(39)
	IgG/IgM/IgA		NR	ELISA	DSM-III	NR	NR	3–12 years	(40)
Neuron-axon filament protein (NAFP)	IgG	200	Bovine	Western blot	DSM-III	54.7% (29/53)	MR 66.6% (8/24) TD 72.4% (16/58)	4–48 years (90% younger than 10 years)	(41)
Glial fibrillary acidic protein (GFAP)	IgG	50	Bovine	Western blot	DSM-III	32% (17/53)	MR 79.2% (5/24) TD 91.4% (5/58)	4–48 years (90% younger than 10 years)	(41)
Ribosomal P protein	IgG/ IgM	17–38	Bovine	ELISA	DSM-IV/CARS	44.3% (31/70)	TD 95% (2.5/48)	4–12 years	(42)
Heat shock protein 90 (Hsp90)	IgG	90	Human cell line	ELISA	NR	19% (4/21)	HC 100% (0/61) AD 100% (0/35)	NR	(43)
Metallothionein (MT)	IgG	6–14	Human	ELISA	NR	32% (13/41)	TD 88% (4/33)	2–16 years	(44)
Glutamic acid decarboxylase 65 (GAD65)	IgG	65	NR	ELISA	DSM-IV	15% (3/20)	ADHD 73% (4/15) TD 100% (0/14)	9–11 years	(45)
Ganglioside M1	NR	1.6	Human	ELISA	DSM-IV	74% (40/54)	TD 95% (2.7/54)	4–11 years	(46)
	NR		Human	ELISA	DSM-V/ADI-R/ADOS/CARS/ABC	37.8% (31/82)	TD 78.3% (13/60)	2–5 years	(47)
Double strand DNA (ds-DNA)	IgG	NR	Human	ELISA	DSM-IV	34% (34/100)	TD 98% (2/100)	4–11 years	(48)
Nucleosome	IgG	NR	Human	ELISA	DSM-IV	46.7% (28/60)	TD 95%	3–12 years	(49)
Nuclear targets (ANA)	IgG	NA	Human	Immunofluorescence	Based on clinical history	27% (3/11)	LKSV 81.8% (2/11)	2–6 years	(50)
	NR	NA	NR	Indirect Immunofluorescence	DSM-IV	20% (16/80)	OND 90% (2/20)	3–12 years	(51)
	NR	NA	NR	Indirect Immunofluorescence	DSM-IV	25% (25/100)	NNI/HC 100% (0/51)	4–11 years	(48)

a *Sensitivity is the percentage of seropositive ASD patients, the amount of seropositive patients over the total tested patient cohort is indicated between brackets*.

b *Specificity is the percentage of seronegativity in the control populations (TD, MR, A, DS, OND, NNI, HC, AD, ADHD, LKSV), the amount of seropositive controls over the total amount of each control cohort is indicated between brackets*.

c *Age range of study population; NA, not applicable; NR, not reported; MR, mental retardation; TD, typically developing; A, adults; DS, Down syndrome; OND, other neurological disorders; NNI, non-neurological illnesses; HC, healthy children; ADHD, attention-deficit/hyperactivity disorder; LKSV, Landau-Kleffner syndrome variant; AD, autoimmune disorder; DSM-III, Diagnostic and statistical manual of mental disorders third edition; DSM-IV, Diagnostic and statistical manual of mental disorders fourth edition; ADOS, Autistic Diagnostic Observation Schedule; ADI-R, Autism Diagnostic Interview-Revised; CARS, Chilhood autism rating scale; ABC, Autism behavior checklist*.

### Protein Targets

#### Myelin Basic Protein (MBP)

Myelin basic protein (MBP) is, together with the proteolipid protein (PLP), the most abundant protein in the myelin membrane (54). It is present on the cytoplasmic side of the membrane in oligodendrocytes of the central nervous system (CNS) and peripheral nervous system (PNS) (55). Its main function is structural, participating in membrane stabilization required for the formation of the myelin sheath, which insulates axons and allows saltatory action potential transmission (56). During brain development, myelination starts in the last weeks of the third trimester of pregnancy, and continues in adult life (57). Myelin integrity is essential for proper cognition, as damage in sheath formation can have severe consequences in the integrity of the white matter networks (58). Alterations in white matter development have been associated with autism manifestations, making MBP a highly interesting protein in the understanding of this effect (10, 59). Moreover, MBP damage can lead to induction of inflammatory and chemotactic mediators which are able to alter the permeability of the BBB (60).

Based on the first descriptions of autoantibodies and abnormal immune function in autistic children (31, 61, 62), Sing et al. (36) investigated the presence of immunoglobulin G (IgG) antibodies against MBP in ASD. MBP autoantibodies had already been described in the CSF of patients with multiple sclerosis (MS) (63). This first study compared a cohort of autistic children under 11 years of age, with age-matched normal children, children with idiopathic mental retardation and children with Down's syndrome (36). MBP-reactive antibodies were found using Western blot in 58% of ASD children, while reactivities in the different control populations were much lower, ranging between 0 and 22% (Table 1). In several follow-up studies, similar or even higher IgG reactivities were reported in autistic children using enzyme-linked immunosorbent assays (ELISA) against MBP (38–40). On the other hand, a study by Libbey et al. (37) could not replicate the presence of these anti-MBP antibodies.

Besides anti-MBP antibodies of the IgG isotype, IgA and IgM antibodies against MBP have also been reported in ASD patients (38, 40). Moreover, autoantibodies of all isotypes were also found against myelin-associated glycoprotein (MAG) and myelin oligodendrocyte glycoprotein (MOG), showing the diversity of the immune reactivity against key myelin proteins in ASD patients. Therefore, these autoantibodies might be informative on the underlying processes of autoimmunity in ASD patients.

However, since anti-MBP antibodies have already been described in several other diseases such as multiple sclerosis, Parkinson's disease, rheumatoid arthritis or schizophrenia (24, 58, 60, 64, 65), it seems unlikely that they would be useful as specific biomarkers for the diagnosis of ASD. Autoantibodies against myelin proteins might still have prognostic value for ASD patients, since serum levels of anti-MBP and anti-MAG antibodies were shown to be increased in severe autism patients compared to patients with mild or moderate disease severity (66).

The reported studies on autoantibodies against MBP in ASD children all have used small patient and control cohorts. Therefore, studies in multiple, independent large cohorts are required using similar detection assays to settle the reported differences in anti-MBP reactivity and to confirm a possible link with disease severity.

#### Neurofilament Protein

Neurofilaments are a class of intermediate filaments, which are part of the neural cytoskeleton, especially in axons of asymmetric, myelinated neurons involved in nerve conduction (67). They are composed of a triplet of proteins with different molecular weights (neurofilament light, 68 kDa; middle, 160 kDa and heavy, 200 kDa) (67). Of these filament proteins, the neurofilament heavy protein (NF-H), also called neuron-axon filament protein (NAFP), is considered as a stable marker for neuron-axon filaments. It has an important role in the development of axons and the transport of vesicles via binding of kinesins, dynein complexes and kinases (68–71). Neurofilament subunit variants have been reported in the development of motor disorders and axonal neuropathies such as amyotrophic lateral sclerosis and Charcot-Marie-Tooth disease (72, 73). Autoantibodies against NF-H have been described in normal aging, but also in degenerative disorders such as Alzheimer's disease, multiple sclerosis and prion diseases (74–77). In ASD, IgG antibodies reactive against bovine NF-H (NAFP) were found using Western blot in nearly 55% of the patients studied (Table 1) (41). However, the percentage of seronegativity in control populations (i.e., specificity) of these autoantibodies is lower than 80% in controls with a typical development and idiopathic mental retardation. A study by Vojdani confirmed the increased presence of IgG, IgM, and IgA autoantibodies against NAFP in autistic patients (40).

#### Glial Fibrillary Acidic Protein (GFAP)

The glial fibrillary acidic protein (GFAP) is a class III intermediate filament cytoskeletal protein. This protein allows the distinction between astrocytes and other glial cells, and is a common astroglial marker in the CNS. Furthermore, it is present in non-myelinating Schwann cells in the PNS and in enteric glial cells (78). In case of acute brain injury, astrocytes become activated, strongly upregulate GFAP expression and increase in size and number, a state called astrogliosis (79). After stroke, traumatic brain injury and spinal cord injury, GFAP, and GFAP breakdown products have been found in CSF and serum (80). These breakdown products trigger an autoantibody response against GFAP in a large subset of patients (81, 82). Elevated GFAP levels have also been described in brain tissue, CSF and serum of ASD patients (83–86), as is the associated autoimmune reactivity. IgG antibodies against GFAP were detected using Western blot in 32% of ASD patients with a specificity of 91% in typically developing subjects, but only 79% in idiopathic mental retarded subjects (Table 1) (41). However, in a small study by Kirkman et al. (87), the average titer of anti-GFAP antibodies in autistic patients was not found to be significantly higher than in controls, raising questions about the role of this antibody-mediated response in this developmental disorder.

#### Ribosomal P Proteins

The ribosomal P proteins are three specific ribosomal proteins located in the large ribosomal subunit (60S). These proteins, P0, P1, and P2, are present in the nucleus and cytoplasm, and are involved in the binding of ribosomal RNA, assembly of the 60S subunit and protein translation (88–90). Autoantibodies reactive against these P proteins are present in nearly one-third of SLE patients and have also been detected in patients with autoimmune hepatitis (91–93). Neuropsychiatric manifestations are some of the most serious complications in SLE, and the occurrence of psychosis in SLE patients has been associated with the presence of anti-ribosomal P antibodies (94). These autoantibodies seem to cross-react with a neuronal integral membrane protein called neuronal surface P antigen (NSPA) (95) and administration of anti-ribosomal P antibodies into murine brains induced neuronal apoptosis and disturbance of the normal functioning of hippocampus and cortex (96, 97), while memory impairment was observed when administered systemically and BBB damage was induced (96). The pathogenicity of these antibodies in other disorders remains unknown, however, in two small studies, elevated anti-ribosomal P protein antibodies were also found using ELISA in 44 to 58% of autistic children and in 5% of the healthy control groups (Table 1) (42, 98). Moreover, the presence of these antibodies in autistic children was related with the levels of the neuropeptide Neurokinin A (42) and with the levels of lead in the blood (98).

#### Heat Shock Protein 90 (Hsp90)

Heat shock protein 90 (Hsp90) is a chaperone involved in assisting the proper folding of proteins and their stabilization (99). Immunoglobulins against this molecular chaperone were found at significantly higher levels in autistic patients than in typically developing controls (43). At the cutoff used, 19% of ASD patients were seropositive for Hsp90 autoantibodies and none of the healthy subjects or controls with autoimmune disorders (Table 1). These reactivities have only been reported for a small study population and have not been replicated in independent studies so far. Therefore, the role of these autoantibodies in autoimmune and other diseases is still unclear.

#### Metallothionein (MT)

Metallothioneins (MT) are intracellular proteins involved in the homeostasis of essential metals and the detoxification of heavy metals (100). The consequences of environmental exposure to noxious agents and their exact role in the pathology of ASD are still under investigation, but it is known that exposure to pollution and heavy metals is causally implicated in changes of fetal neurodevelopment that are related with autism (8, 101, 102). The presence of anti-MT antibodies has been described in metal-induced diseases such as occupational heavy metal exposure and metal allergy (103, 104). Furthermore, one in four autistic children showed a higher MT concentration in the serum and one third of ASD patients showed increased levels of autoantibodies against MT (Table 1) (105). These findings might indicate immune abnormalities related to levels of metal exposure in ASD patients.

#### Glutamic Acid Decarboxylase (GAD65)

GAD65 is the enzyme responsible for the conversion of glutamate to GABA, an inhibitory neurotransmitter in the CNS (106). Anti-GAD65 antibodies have typically been associated with autoimmune diseases not related with the nervous system such as type 1 diabetes, however they have also been reported in CNS disorders such as encephalitis, epilepsy and cerebellar ataxia, where they could even have a pathological role (106–110). The role of GAD65 in neurotransmitter production and the relationship with seizure episodes made it an interesting target in brain disorders with excitation/inhibition imbalance such as autism (106). In a small study using 20 patients, the presence of anti-GAD65 antibodies measured using ELISA was reported in 15% of the autism cases, 27% of ADHD patients and in none of the controls (Table 1) (45). Despite the size of the study, an initial indication of a possible relation between these autoantibodies and autism was made. In the future, it might be interesting to test the presence of anti-GAD65 antibodies in the subpopulation of ASD patients that present ataxia-related problems or seizures.

### Non-protein Targets

#### Ganglioside M1 (GM1)

Ganglioside M1 is a glycosphingolipid with one sialic acid residue that is present in the cell membrane (111). It is involved in neuronal plasticity and neurotrophin secretion during development, adulthood and aging (111, 112). Anti-GM1 antibodies have been described in motor neuropathies, but also in neurodegenerative diseases such as GM1 gangliosidosis, multifocal motor neuropathy, dementia and Guillain-Barré syndrome (113–116). Two studies have reported increased levels of these antibodies in ASD patients using ELISA, however with widely different sensitivities, varying from 74 to 38%, and corresponding specificities of 95 and 78%, respectively (Table 1) (46, 47). Moreover, in the study by Mostafa et al. (46), a significant correlation with disease severity was found, whereas this could not be confirmed in another study (47). Nevertheless, it seems that anti-GM1 antibodies have been associated with ASD, but their specific role remains to be elucidated.

#### Nuclear Antigens

Antinuclear antibodies (ANAs) are immunoglobulins that react against nuclear components, mostly double stranded DNA (dsDNA) and DNA-associated proteins. These antigens are not organ-specific and are ubiquitous in every cell. ANAs have been detected in cancer and premalignant diseases, but mostly in autoimmune disorders such as SLE (117–119). Renal failure, nephritis and damage in the glomerular structures have been related to these antibodies (120–123). ANAs, anti-dsDNA and anti-nucleosome antibodies have also been described in autism patients (48–51). Immunoglobulins against general nuclear targets were found in up to a quarter of ASD patients using indirect immunofluorescence detection (Table 1) (48, 49, 51). These ANAs show a high specificity (over 96%) in typically developing children. More specific measurements, using ELISA for anti-ds-DNA or anti-nucleosome antibodies, showed that, respectively 34 and 47% of ASD patients were seropositive for these ANAs (48, 49). The presence of antinuclear immunoglobulins has been reported to be increased in patients with severe autism, but also with abnormal electroencephalogram results and mental retardation, suggesting that even if these antibodies cannot be used as a diagnostic test, they might be symptom related (50, 51). In addition, the presence of these antibodies is related with a family history of autoimmunity, even more related to autoimmune diseases in female relatives (51). Between 29 and 48% of the ASD patients seropositive for anti-nuclear antibodies have an autoimmune background (48, 51). The presence of high titers of anti-nuclear antibodies in other autoimmune disorders reduces the chances of using ANAs patterns as a separate diagnostic tool in autism (50, 124).

## Maternal Autoantibodies

After being born from the sterile environment of the womb, neonates are rapidly exposed to many different microbial and environmental antigens, while their immune system is still inexperienced. Therefore, during the first months of life, an important mechanism of protection is provided via passive immunization with antibodies, which are been transferred from the mother to the fetus during pregnancy [reviewed in Palmeira et al. (125)]. This occurs via binding to the neonatal Fc receptor (FcRn), which transports IgG subclasses 1, 3, and 4 across the placenta, during the second and third trimester of pregnancy.

In the nineties, around the same time the involvement of the immune system and the presence of autoantibodies in ASD patients were being elucidated, the relationship between aberrant maternal immune responses and autism in children was also being explored. The history of spontaneous abortions and disorders during pregnancy in mothers that later had a child with ASD, together with the suggestion of the presence of anti-paternal antibodies in mothers with a complicated pregnancy record, led to the study of antibody reactivity of maternal plasma against targets in their children (126). In this context, Warren et al. detected increased complement-dependent cytotoxicity by maternal antibodies in 54% (6/11) of mothers of autistic children and in 10% (2/20) of mothers of normal children (126). Despite the small number of subjects, this study was the first indication of a possible correlation between maternal antibodies and the development of autism in their children. Binding of maternal antibodies to brain proteins was first described in a case study (30). Using serum from a mother of 2 children with different developmental disorders including autism, binding of autoantibodies to Purkinje cells and large neurons in murine cerebellum and brainstem was described. A direct functional role for these antibodies was also suggested, as passive transfer of serum of this mother in pregnant mice led to altered exploratory behavior in the pups (30).

In order to further understand this reactivity, several studies were performed to identify maternal antibodies and their antigenic targets (Table 2). In a study by Zimmerman, 45% (5/11) of mothers of autistic children showed reactivity against a pattern of 5 proteins between 15 and 37 kDa of fetal rat brain using Western blot (130). This pattern was not observed in mothers of typically developing children or using postnatal and adult rat brain extracts. Using a bigger cohort of 100 mothers of children with autistic disorder and 100 controls, immunoreactivity using Western blot against a 36 kDa human fetal brain protein was found in 10% of the mothers of ASD children (MASD) and only in 2% of the mothers with typically developing children (MTD) (128). A 36 kDa and a 73 kDa band were also identified using rodent embryonic brain tissue in 47–48% of MASD with a specificity of 69% in MTD. In addition, the group of Van de Water performed multiple analyses on maternal samples from the CHARGE study (Childhood Autism Risks from Genetics and the Environment), where mothers were sampled up till 5 years after delivery, but also on maternal mid-pregnancy serum samples from the EMA study (Early Markers for Autism) (131–133, 135, 136). Highly specific patterns of immunoreactivity toward rhesus macaque or human fetal brain extract were found in mothers of children with autism, where a 73 kDa band was combined with either a 37 or a 39 kDa band. Mass spectrometric analysis of immunoreactive spots from a fetal rhesus macaque brain protein extract, resulted in the identification of seven primary targets of maternal antibodies related with autism: lactate dehydrogenase A and B (LDH-A/B) (37 kDa), Y-box-binding protein 1 (YBX1) (39 kDa), Cytosolic PSD-95 interactor (Cypin) (44 kDa), Collapsin response mediator protein 2 (CRMP2) (62 kDa), Collapsin response mediator protein 1 (CRMP1) (70 kDa), and Stress-induced phosphoprotein 1 (STIP1) (73 kDa) (Table 2) (127). Individually, testing immunoreactivity against these targets using Western blot resulted in sensitivities ranging from 18 to 59% in mothers of ASD children, with specificities between 64 and 93% in mothers of typically developing children (Table 2). Combination of different panels, each consisting of immunoreactivity against specific combinations of two or more antigens of these seven targets, led to a large increase in specificity (99%), while maintaining a relatively high sensitivity of 23% (56/246) (Table 2). Moreover, an increased impairment in stereotypical behavior was observed in children of mothers with LDH-, or combined LDH/STIP1- or LDH/STIP1/CRMP1 immunoreactivity.

**Table 2:** Overview of maternal autoantibodies in ASD.

**Maternal autoantibodies**								
**Antibody target**	**Isotype**	**MW (kDa)**	**Protein Origin**	**Methods**	**Sensitivity^a^**	**Specificity^b^**	**Serum/Plasma**	**References**
Lactate dehydrogenase A and B (LDH)	IgG	37	Rhesus macaque brain (152 days)	Western blot	28% (68/246)	MTD 87% (20/149)	Plasma	(127)
Cytosolic PSD-95 interactor (Cypin)	IgG	44	Rhesus macaque brain (152 days)	Western blot	25% (62/246)	MTD 81% (29/149)	Plasma	(127)
Stress-induced phosphoprotein 1 (STIP1)	IgG	73	Rhesus macaque brain (152 days)	Western blot	59% (145/246)	MTD 64% (53/149)	Plasma	(127)
Collapsin response mediator proteins 1 (CRMP1)	IgG	70	Rhesus macaque brain (152 days)	Western blot	32% (78/246)	MTD 82% (27/149)	Plasma	(127)
Collapsin response mediator proteins 2 (CRMP2)	IgG	62	Rhesus macaque brain (152 days)	Western blot	18% (44/246)	MTD 93% (11/149)	Plasma	(127)
Y-box-binding protein (YBX1)	IgG	39	Rhesus macaque brain (152 days)	Western blot	31% (78/246)	MTD 77% (34/149)	Plasma	(127)
LDH + STIP1 + CRMP1	IgG	37 +73	Rhesus macaque brain (152 days)	Western blot	5% (13/246)	MTD 100% (0/149)	Plasma	(127)
LDH + STIP1 + CRMP1 + Cypin	IgG	NR	Rhesus macaque brain (152 days)	Western blot	2% (5/246)	MTD 100% (0/149)	Plasma	(127)
Specific combinations of LDH, STIP1, CRMP1, Cypin, CRMP2, YBX1	IgG	NR	Rhesus macaque brain (152 days)	Western blot	23% (56/246)	MTD 99% (2/149)	Plasma	(127)
Purkinje cells	IgG	NR	Adult rat brain P1 mouse brain	Immunohistochemistry	Case study	Case study	Serum	(30)
NB-1 neuroblastoma	IgG	NR	Human cell line	Immunohistochemistry	Case study	Case study	Serum	(30)
GFAP	IgG		Human	Western blot	10% (2/20)	MTD 85% (3/20)	Serum (years after delivery)	(128)
MBP	IgG	18–20	Human	Western blot	5% (1/20)	MTD 90% (2/20)	Serum (years after delivery)	(128)
Nuclear targets (ANA)	IgG	NR	Mouse (12 weeks)	Immunohistochemistry	24.5% (251/1022)	MTD 84.9% (52/345)	Plasma	(129)
Fetal Brain proteins	IgG	15–37 >250	Fetal rat brain	Western blot	45.4% (5/11) 54.5% (6/11)	MTD 100% (0/10) MTD 100% (0/10)	Serum	(130)
	IgG	36	Human (17-weeks)	Western blot	10% (10/100)	MTD 98% (2/100)	Serum (years after delivery)	(128)
	IgG	37	Human (20–40 weeks)	Western blot	26.2% (16/61)	MTD 91.9% (5/62) MDD 97.5% (1/40)	Plasma (2–5 years after delivery)	(132)
	IgG		Rhesus macaque brain (152 days)	Western blot	6.6% (17/259)	MTD 93.9% (11/180)	Plasma	(132)
	IgG	39	Human (20–40 weeks)	Western blot	7.1% (11/84)	MTD 98% (3/152) MDD 100% (0/48)	Serum (15–19 weeks of gestation)	(131)
	IgG		Human (17-weeks)	Western blot	14% (14/100)	MTD 85% (15/100)	Serum (years after delivery)	(128)
	IgG		Rhesus macaque brain (152 days)	Western blot	10.4% (27/ 259)	MTD 83.9% (29/180)	Plasma	(132)
	IgG	60	Human (20-40 weeks)	Western blot	19% (16/84)	MTD 76.3% (36/152) MDD 75% (12/48)	Serum (15–19 weeks of gestation)	(131)
	IgG	61	Human (17-weeks)	Western blot	30% (30/100)	MTD 69% (31/100)	Serum (years after delivery)	(128)
	IgG	73	Human (20–40 weeks)	Western blot	13.1% (11/84)	MTD 92.8% (11/152) MDD 95.8% (2/48)	Serum (15–19 weeks of gestation)	(131)
	IgG		Rhesus macaque brain (152 days)	Western blot	16.2% (42/259)	MTD 87.8% (22/180)	Plasma	(132)
	IgG	37 + 73	Human (20–40 weeks)	Western blot	11.5% (7/61)	MTD 100% (0/62) MDD 100% (0/40)	Plasma (2–5 years after delivery)	(133)
	IgG		Monkey brain	Western blot	9% (9/202)	MTD 100% (0/163)	Plasma	(134)
	IgG		Rhesus macaque brain (152 days)	Western blot	9.3% (24/259)	100% (0/180)	Plasma	(132)
	IgG		Rhesus macaque brain (49, 100, 152 days)	Western blot	7% (10/143)	MTD 100% (0/121) MDD 100% (0/62)	Plasma	(135)
	IgG		Rhesus macaque	Western blot	7.6% (10/131)	MTD 100% (0/50)	Plasma	(136)
	IgG	39 + 73	Human (20-40 weeks)	Western blot	3.6% (3/84)	MTD 100% (0/152) MDD 95.8% (2/48)	Serum (15–19 weeks of gestation)	(131)
	IgG		Rhesus macaque brain (152 days)	Western blot	8.9% (23/259)	MTD 98.3% (3/180)	Plasma	(132)
	IgG	60 + 73	Human (20–40 weeks)	Western blot	6% (5/84)	MTD 98% (3/152) MDD 97.9% (1/48)	Serum (15–19 weeks of gestation)	(131)
	IgG	NR	Mouse (12 weeks)	Immunohistochemistry	10.7% (260/2431)	MTD 97.4% (17/653)	Plasma	(129)
Adult Brain cingulate gyrus protein	IgG	91 100 129	Human	Western blot	13 % (13/100) 5% (5/100) 26% (26/100)	MTD 80% (20/100) MTD 100% (0/100) MTD 79% (21/100)	Serum (years after delivery)	(128)
Adult Brain cerebellum protein	IgG	31 100	Human	Western blot	25% (25/100) 34% (34/100)	MTD 65% (35/100) MTD 70% (30/100)	Serum (years after delivery)	(128)
Adult Brain caudate protein	IgG	81 155	Human	Western blot	20% (20/100) 11% (11/100)	MTD 79% (21/100) MTD 98% (2/100)	Serum (years after delivery)	(128)
Adult Brain frontal cortex (BA9)	IgG	63	Human	Western blot	39% (39/100)	MTD 73% (27/100)	Serum (years after delivery)	(128)
Embryonic tissue protein	IgG	36 73	Rat (E18)	Western blot	48% (48/100) 47% (47/100)	MTD 69% (31/100) MTD 69% (31/100)	Serum (years after delivery)	(128)
Adult Brain protein	IgG	27 110	Rat	Western blot	22% (22/100) 29% (29/100)	MTD 90% (10/100) MTD 66% (34/100)	Serum (years after delivery)	(128)

a *Sensitivity is the percentage of seropositive mothers of ASD children, the amount of seropositive mothers of ASD children over the total tested cohort of mothers of ASD is indicated between brackets*.

b *Specificity is the percentage of seronegativity in the control populations (MTD, MDD), the amount of seropositive controls over the total amount of each control cohort is indicated between brackets. NR, non-reported; MASD, mother of children with autism spectrum disorder; MTD, mother of children with typically developing; MDD, mother of children with non-autistic developmental delay*.

The active contribution of these maternal autoantibodies in the development of autistic features in the offspring has been investigated in animal models using passive transfer of the total IgG fraction purified from mothers of ASD children or mothers of typically developing children. Pregnant mouse dams have been injected with purified IgG around the end of the second trimester of pregnancy, either with a single injection in the maternal periphery (137) or in the embryonic brain ventricles (69, 138), or by daily peripheral injections during the third trimester (139). Recently, a novel active immunization model has also been applied, where female mice were immunized before pregnancy with a mixture of immunogenic peptides from LDH-A/B, STIP1, and CRMP1, providing a more representative situation where mouse dams produce antibodies themselves during the entire pregnancy (140, 141). Mice exposed to these maternal autoantibodies during development showed increased cell division of radial glial cells in the subventricular zone, increased neuronal size in the cortex (142) and a reduction in the number of dendritic spines and of the dendritic arborization in the neurons of the infragranular layers of the adult cortex (138). This coincides with autism-related behavioral changes such as impaired motor and sensory development, hyperactivity, anxiety, inappropriate social interactions and repetitive and stereotypic behaviors (69, 137, 139, 141). While these maternal autoantibodies have been shown to reach high levels in the brains of the embryos, only a very low amount could be found in the brains of the dams (137, 139), probably due to differences in BBB permeability in embryonic and adult brains. Moreover, behavioral changes in the dams themselves have not been reported so far.

These studies do not directly pinpoint the involvement of a single autoantibody target in ASD development, but remain an important tool to experimentally demonstrate that autoantibody transfer during pregnancy can alter brain biology, resulting in autistic behaviors in the offspring. A detailed overview of the autoantibodies described in mothers of children with ASD is presented in Table 2. The biomarker potential, general function, expression and possible role during neurodevelopment of the most interesting autoantibody targets is discussed below in more detail.

### Lactate Dehydrogenase A and B (LDH-A and LDH-B)

Antibodies to LDH-A and LDH-B were found in 28% of mothers of ASD children and 13% of mothers of typically developing children (Table 2) (127). Lactate dehydrogenase (LDH) is an oxidoreductase responsible for the reversible conversion of the glycolytic intermediate pyruvate to lactate. This leads to rapid energy production via fermentation of pyruvate, instead of fully metabolizing it to CO_2_ via aerobic respiration in the mitochondria. LDH is encoded by 3 different genes *LDH-A, B* and *C* that correspond to 3 different protein subunits, which can combine into homotetrameric or heterotetrameric isoenzymes (143). LDH-A and LDH-B are differentially expressed in different adult brain regions, with LDH-A showing a diffusely distributed expression pattern, with increased expression in the hippocampus and cortex, while LDH-B shows high expression in several brain structures such as the olfactory bulb and piriform cortex, several thalamic and hypothalamic nuclei, the granular, and Purkinje cell layers of the cerebellum and a laminar pattern in the neocortex (144). Isoenzymes which are preferentially composed of LDH-A or LDH-B have different metabolic functions, and the ratio between A and B is responsible for the balance between glycolysis and oxidative phosphorylation in different tissues (143).

Serum LDH activity has been found to be increased in ASD patients and appears to be related to the level of disease severity (145). Interestingly, increased levels of pyruvate and lactate, the metabolites which are interconverted by LDH, are often used as a marker for mitochondrial dysfunction, as less pyruvate is metabolized through the tricarboxylic acid cycle in mitochondria [reviewed in Rossignol and Frye (146)]. Besides increased levels of pyruvate and lactate, several other lines of evidence, such as altered levels of other carbon metabolites and activity changes or mutations in enzymes involved in carbon metabolism, indicate a broader mitochondrial dysfunction in a subset of autism patients (147, 148). As neurons are highly energy dependent cells, dysregulation of energy metabolism can implicate changes in their main functions and structures. Still, the exact role of mitochondrial dysfunction and carbon metabolism in ASD pathology remains poorly understood.

### Y-Box-Binding Protein 1 (YBX1)

Antibodies to YBX1 were found in 31% of mothers of ASD children and 23% of mothers of typically developing children (Table 2) (127). Specific combinations of antibody reactivity to YBX1 and LHD or CRMP2 were found in a smaller percentage of mothers of ASD children (2–5%), but with much higher corresponding specificities (99–100%). *YBX1* encodes the Y-box-binding protein 1, also called nuclease-sensitive element-binding protein 1. This protein is involved in DNA and RNA-binding during mRNA processing, splicing, transcription and transcription regulation and is important for processes such as stress response, cell proliferation, and differentiation (149, 150). Furthermore, this protein is involved in differentiation of neuronal progenitors, maintenance of the stem cell status and malignant cell transformation (151). YBX1 has a general expression in most murine tissues, both during embryonic development and in adulthood (152). In the adult human brain, YBX1 is mainly located in neurons throughout the brain, while prenatally it is expressed in radial glia, neuroblasts and neurons (153). Homozygous knockout of *YBX1* leads to embryonic lethality, with abnormalities in neural tube formation (154).

The role of YBX1 in ASD is still unknown, but it has a direct protein-protein interaction with methyl-CpG binding protein 2 (MeCP2), which is related with neurodevelopmental disorders such as Rett syndrome (155). This interaction between YBX1 and MeCP2 is dependent on RNA, and influences RNA splicing. Furthermore, after maternal immune activation, the expression of YBX1 is rapidly increased in the fetal brain, however, the possible role in adverse neurological outcomes has not been studied in this context (156).

### Cytosolic PSD-95 Interactor (Cypin)

Mothers of autistic children showed antibody reactivity against Cypin, in 25% of cases, compared to 19% in mothers of typically developing children (Table 2) (127). Cypin is a guanine deaminase, which is required for the enzymatic degradation of the purine guanine to xanthine (157). Cypin is expressed in various organs in the body, but its highest expression is seen in epithelial cells of the intestine and in discrete neurons in the cortex, hippocampus and the superior colliculus (158). Importantly, Cypin has been found to be a major interaction protein for postsynaptic density protein 95 (PSD-95), and has both a structural and a regulatory role, decreasing localization of PSD-95 at post-synaptic sites (158). Cypin also promotes microtubule polymerization by directly binding to tubulin using a domain with high homology to collapsin response mediator protein (CRMP), another maternal autoantibody target described in autism (127, 159). This CRMP-homology domain and a 9 amino acid zinc-binding domain are required for guanine deaminase activity and for the regulation of dendrite branching and neuronal morphology in cultured hippocampal neurons (159, 160). Dendrite morphogenesis is essential for the communication between neurons and when the regulation of the dendritic arborization is altered, this can lead to abnormal spine density and morphology, synapse loss and aberrant synaptic signaling, resulting in modification of the neural circuitry which can lead to neuropsychiatric disorders (14, 161, 162).

### Collapsin Response Mediator Proteins 1 and 2 (CRMP1/CRMP2)

Antibodies to CRMP1 or CRMP2 have been found in 32 and 18% of mothers of ASD children, respectively, and in 18 and 7% of mothers of typically developing children (Table 2) (127). In addition, these autoantibodies are also found together with many of the other described autoantibodies in mothers of autistic children. The collapsin response mediator proteins are a family of five phosphoproteins (CRMP1-5) located in the cytosol of both neurons and glia (163). CRMP1 and CRMP2 are expressed both in the developing and the adult brain, but show peak expression in the last weeks of gestation and the first postnatal weeks (164). These proteins participate in the organization of the cytoskeleton and bind to microtubuli, actin filaments and intermediate filaments (165–168). During cell division, CRMP1 and 2 stabilize the mitotic apparatus, by binding to microtubuli (166). CRMP1 and CRMP2 are involved in radial migration and the subsequent differentiation of neurons during cortical development (169, 170). Furthermore, both proteins play a role in the regulation of signaling of class 3 semaphorins and neural growth cone collapse, remodeling of the cytoskeleton required for axonal growth and guidance, and dendritic arborization (171–174). During neuronal polarization of cultured hippocampal neurons, CRMP2 becomes highly enriched in the distal part of the growing axon (172). On the other hand, when CRMP2 is overexpressed, these neurons develop multiple axons, while the expression of a truncated dominant negative mutant resulted in neurons with short or no axons. In addition, CRMP1 and CRMP2 are also required for proper dendritic patterning of cortical neurons. CRMP1^−/−^ mice show reduced dendrite length and reduced dendritic spine density, while CRMP2^−/−^ mice only have a reduced spine density (174). Both proteins seem to have some functional redundancy as inhibition of CRMP2 phosphorylation in a CRMP1^−/−^ background induces a strong disruption of the morphology, orientation and guidance of the dendrites of layer V cortical pyramidal neurons (175).

CRMP1^−/−^ mice showed impaired memory and behavioral abnormalities related to schizophrenia such as hyperactivity, altered emotional behavior and decreased prepulse inhibition (176). On the other hand, brain-specific knockout of CRMP2 induced impairment of learning, memory and social behavior (177), while knock-in of a CRMP2 mutant with impaired phosphorylation reduced emotional behavior, sociality and pain sensitivity (178). Multiple genome-wide and proteome-wide analyses have described gene variants or altered levels of CRMP1 and 2, but also other CRMP family members, in patients with neurodegenerative and neuropsychiatric disorders, such as Alzheimer disease, schizophrenia, mood disorders, epilepsy and neuropathic pain [reviewed in Quach et al. (179)].

### Stress-Induced Phosphoprotein 1 (STIP1)

Mothers of ASD children showed antibody reactivity against STIP1, in 59% of cases, compared to 36% in mothers of typically developing children (Table 2) (127). In addition, anti-STIP1 antibodies were also found in many combinations with the other described maternal autoantibodies, resulting in strongly increased specificity. The Stress-induced phosphoprotein 1 (STIP1) is a co-chaperone of the heat shock proteins (Hsp)70 and Hsp90, and modulates their folding activity (180). STIP1 is mostly expressed in the cytosol, but it can also translocate to the nucleus (181), and even be secreted in exosomes, where it is found on the surface (182). Extracellular STIP1 was found to interact with the glycosylphosphatidylinositol (GPI)-anchored cellular prion protein PrP^C^, leading to an increased number of neurons with neurites *in vitro*, without affecting the number or length of neurites per cell (183, 184). Moreover, this STIP1-PrP^C^ interaction also protected cultured neurons from staurosporine-induced cell death. This STIP1-PrP^C^-induced neuritogenesis and neuroprotection was found to be mediated by Ca^2+^ signaling through the alpha7 nicotinic acetylcholine receptor (185). Interestingly, during glutamate-induced neurogenesis *ex vivo*, STIP1 and CRMP2 were among the most upregulated proteins (186). Moreover, *STIP1*^−/+^ heterozygous mice, which have a 50% reduction of STIP expression without affecting Hsp70, Hsp90 or the PrP^C^ levels, showed hyperactivity and attention deficits (187).

### Contactin-Associated Protein-Like 2 (CASPR2)

Contactin-associated protein-like 2 (CASPR2) is a transmembrane cell adhesion protein of a subgroup of the neurexin family related to the establishment of the neural network and higher cognitive functions in the brain (188). The protein is encoded by the *CNTNAP2* gene and is expressed both in the developing and adult nervous system (189, 190). In the developing human cortex, the anterior temporal and prefrontal regions have shown specific enrichment of CASPR2 (190). In adults, it is expressed in areas of the limbic circuit and in brain areas involved in motor activities and sensory pathways (191). At the subcellular level CASPR2 is located at the axon initial segment and in the nodes of Ranvier of myelinated axons, in presynaptic terminals of inhibitory neurons and in the postsynaptic compartment of excitatory neurons (189, 192). It is implicated in processes as diverse as nerve excitation and conduction, neurotransmitter release, organization of the axonal domain, dendritic spine stabilization, and neuronal network formation (193, 194).

Copy number variations and single nucleotide polymorphisms in the *CNTNAP2* gene have been related to several neurodevelopmental disorders, such as schizophrenia, intellectual disability, epilepsy and autism, often with combined clinical presentation [reviewed in Saint-Martin et al. (188); Poot (195)]. Interestingly, genetic knockout of this gene in a mouse model induces the development of many of the core ASD behavioral symptoms, as *CNTNAP2*−/− mice show increased repetitive behavior, decreased vocalization and decreased social interactions (196). In addition, these mice also display hyperactivity, hyper-reactivity to thermal sensory stimuli and epileptic seizures and allowed to study some of the underlying biology, with observed abnormalities in neuronal migration, a reduction in GABAergic interneurons and abnormal neuronal network activity.

In addition to the genetic variants affecting CASPR2 function, acquired dysfunction of CASPR2 has also been proposed in patients that form autoantibodies against CASPR2. Such antibodies have been described in patients with limbic encephalitis, Morvan syndrome and neuromyotonia, three conditions that variously affect the central and peripheral nervous system, and are characterized by cognitive decline, epilepsy, peripheral nerve hyperexcitability and neuropathic pain (197–199). Patient CASPR2 autoantibodies decreased AMPA-type glutamate receptor trafficking in cultured neurons and perturbed cortical excitatory transmission after stereotactic injection in the mouse visual cortex (200). Systemically injected patient CASPR2 autoantibodies did not cross the BBB and caused peripheral hypersensitivity to mechanical stimuli (201).

In contrast, *in utero* exposure to CASPR2 autoantibodies during embryonic development has been linked to the development of autistic features in the offspring (202). A monoclonal antibody with reactivity to CASPR2 was cloned from the memory B cells of a mother with brain-reactive serology and a child with autism, and used in a passive transfer experiment in pregnant mice (202). The male offspring showed abnormal cortical development, together with a reduction in the dendritic complexity in excitatory neurons and a reduced number of inhibitory neurons, and presented with repetitive behaviors and impairment in learning and social abilities. In addition, purified human IgG from patients with CASPR2 immunoreactivity, could be found in the fetal circulation and fetal brain parenchyma after injection into pregnant mice (203). These offspring also showed deficits in social interaction, together with abnormal neuronal distribution, decrease in excitatory synapses and an increase in microglial activation (203). Among mothers of an autistic child that show brain-reactive antibodies, 37% were found to have autoantibodies that bind to CASPR2, compared to 12% in mothers of an autistic child lacking brain reactive antibodies and 8% of women of a normally developing child (202).

## Conclusion

In conclusion, an increasing number of ASD-related autoantibodies have been described, both in ASD patients themselves and in mothers with children that later develop ASD.

At the moment, most of the antibodies which have been described in ASD patients still lack validation between independent research groups, and require testing in higher numbers of ASD patient and relevant control samples using standardized assays for a more reliable determination of their possible value as disease biomarkers. These autoantibodies have mostly been tested using a candidate approach, using autoantibodies which have already been described in other autoimmune diseases. This field might benefit from an unbiased screening of immunoreactivity in ASD patients, to identify potential new autoantibodies with a higher specificity for ASD. Because of the lack of functional studies using passive transfer of ASD patient antibodies in animal models, it is not known whether autoantibodies in ASD patients have an active contribution to the initiation or worsening of the disease. It could be that the presence of these antibodies is not directly related with the disease, considering the high prevalence in other disorders. Still, they might have indirect roles in the pathology or be related with subpopulations of ASD patients.

Autoantibodies in mothers of children that later develop ASD, have been found using a screening of immunoreactivity against relevant brain tissue, or in the case of CASPR2, by studying a protein which has already shown high relevance for ASD, and for which autoantibodies were previously described in other disorders. Individual specificities of these maternal antibodies are low, which could only be significantly increased using certain combinations of maternal antibody reactivity. These candidate markers also require validation by independent research groups, especially considering the specific set of combinations that have been described. However, an active contribution of certain autoantibodies in mothers, to the development of ASD in their children, is gaining more evidence. Passive transfer of IgG from mothers of ASD children in pregnant mice, or LDH-A/B, STIP1- and CRMP1-immunization of female mice leads to relevant ASD-related biological and behavioral changes in the offspring. Individual relations of these autoantibodies to specific biological processes have not been established, and quite possibly, a combination of autoantibodies is required for a clear behavioral effect. Still, these are compelling models to further study the details of autoantibody-mediated disruption of early processes of neurodevelopment during pregnancy, eventually leading to behavioral deficits linked to ASD later in life.

## Author Contributions

RM-C wrote the manuscript and designed the figure and tables. PV wrote the manuscript, reviewed the manuscript structure, ideas, and science. VS evaluated and reviewed manuscript structure, ideas, and science. All authors read and approved the final manuscript.

### Conflict of Interest Statement

The authors declare that the research was conducted in the absence of any commercial or financial relationships that could be construed as a potential conflict of interest.
